# Procedures by Physician Associates in Obstetrics and Gynecology

**DOI:** 10.1089/whr.2023.0044

**Published:** 2023-11-16

**Authors:** Melissa A. Rodriguez, Roderick S. Hooker

**Affiliations:** ^1^ Orlando Health Winnie Palmer Hospital for Women and Babies, Orlando, Florida, USA.; ^2^ Retired.

**Keywords:** physician associate, obstetrics, gynecology, workforce, procedures

## Abstract

**Background and Objectives:**

The number of obstetricians and gynecologists in the United States is decreasing and providers backfilling this service have not been well described. The intent of the study was to identify the skills that physician associates (PAs) in obstetrics and gynecology (OBGyn) contribute to this aspect of medicine and surgery.

**Methods:**

A survey of PAs specializing in OBGyn was conducted in 2022. The intent was to list office-based procedures that were part of their skill set. A vetted questionnaire was sent to the 1,630 American Academy of Physician Associates members who identified themselves in OBGyn at some point in their career, and 729 responded (44.7% relative risk).

**Results:**

Most PAs (88.7%) in OBGyn first assist in surgery. This first-assist role ranged across the open, laparoscopic, and robotic-type operations. Categories of surgery included Cesarean section, hysterectomy, salpingo-oophorectomy, and subspecialty surgeries such as oncology and urogynecology. In the outpatient setting, PAs listed over 40 procedures ranging from biopsies of the endometrium, cervix, vagina, and vulva, as well as fetal assessment, ultrasonography, and long-acting contraceptive insertion and removals.

**Conclusions:**

The proceduralist role of PAs in OBGyn is broad. Furthermore, this role may need to be utilized more at a time of growing scarcity of clinicians. The OBGyn role for PAs adds to their specialization and increasing presence in American medicine.

## Introduction

The US health care system requires more clinicians across various medical and surgical practices.^1^ In obstetrics and gynecology (OBGyn), the problem is particularly acute. Nationally, the *Health Resources and Services Administration* (HRSA) predicts the number of physicians providing OBGyn services will decline from an estimated 50,850 FTEs in 2018 to an estimated 47,490, or 3,360 fewer, by 2030.^2^ The reasons for the waning of critical health care services are multifactorial and include potential generational and gender differences in employment expectations.^3^ Concurrently, certified nurse midwives (CNMs) and nurse practitioners (NPs) in women's health are expected to increase to a national workforce of 10,260 and 20,020, respectively, during this same period.^2^ Family medicine doctors provide some degree of women's health, which is declining.^4^

Additionally, 73.1% of obstetrician-gynecologists and 72.6% of nurse midwives prescribed oral contraceptives, patches, or rings. Yet only 51.4% of family medicine physicians, 32.4% of pediatricians, and 19.8% of internal medicine physicians do so.^5^ These low numbers occur at a time when access to women's health is constrained.

The HRSA 2021 forecast for physician associates (PAs) in OBGyn during this period was 1,480 and is estimated to grow to 1,530 by 2030.^2^ The 2021 National Commission on the Certification of PAs (NCCPA) supplemented this number with a census of certified PAs in OBGyn at 1,322 (1.2% of all clinically active PAs) in 2021.^6^

As background, the NCCPA database report contains information on all certified PAs and spans 70 medical and surgical disciplines.^7^ PA medical education and training are intensive over three academic years.^8^ Didactic courses include basic medical science, clinical medicine, and behavioral sciences. The clinical phase consists of over 2,000 hours of supervised clinical practice experiences in medical and surgical disciplines, including OBGyn. The PA OBGyn population has increased by 67% since 2013 when only 792 PAs were practicing in this specialty.^9^ In 2021, the median age was 38 years, and 98% female. In comparison, 70.1% of PAs in all 70 PA specialties were female. The practice settings of PAs in women's health care are outpatient offices (54.7%) and hospitals (34%), with 11.3% described as “other.”^7^ Outpatient PAs manage diverse gynecological conditions, perform office-based procedures, preventative care screenings, family planning, infertility care, and sexual health, and manage pregnancy from the first trimester through childbirth and postpartum.

Inpatient PAs provide care in a hospital or work as hospitalists in obstetric and gynecologic services. Gynecologic procedures include long-acting contraceptive insertion and removal, colposcopy, cryotherapy, insemination, endometrial and vulvar biopsies, loop excision electrocautery procedure, and first-assist in surgery. In obstetrics, PAs manage labor, perform vaginal deliveries, assist with vacuum and Cesarean deliveries, and manage obstetric emergencies such as postpartum hemorrhage. Additionally, PAs integrate well with physician resident services to support the resident's education and provide continuity of care and timely patient discharges.^10^

Because the projected shortage of physicians in OBGyn is a looming issue in national health workforce forecasts, we set out to understand the PA's role in women's health service delivery. The objective is to broaden the knowledge base about PAs' skillset in medicine and surgery. Medical providers that provide cancer screening procedures, contraceptive access, and whole-pregnancy care are in high demand, and PAs routinely provide this care.

## Methods

Common procedures in OBGyn were drawn from the Association of Physician Associates in Obstetrics and Gynecology (APAOG) OBGyn PA Core Competencies document. The authors developed a mixed methods survey instrument, piloted it with five APAOG members, and retested it. The survey was reformatted for quick replies to 30 questions, algorithm-driven, and timed to take 15 minutes on average to complete. Open text boxes followed each question for voluntary input. The final questionnaire was sent electronically *via* Qualtrics© Core XM, a surveying and marketing research company, to all 1,630 Academy of Physician Associates (AAPA) members that identified their PAs role as OBGyn. Participation was voluntary. Two notices were sent (electronically and by newsletter) stating that the Orlando Health Institutional Review Board had approved the anonymous survey.

The introduction letter for the survey identified that this study was a priority set by the APAOG Board of Directors. No incentives or enticements were offered. Five reminders of the survey were sent 2 weeks apart after the survey was launched; the survey closed in December 2022. Quantitative results were reported using descriptive statistics. Qualitative results were reported as a thematic analysis utilizing *Dovetail* (https://dovetail.com), a research repository tool for centralizing interview notes with tagging and annotations to help with analysis.

## Results

In total, 729 PAs in OBGyn responded to the anonymous survey by answering fully or in part (47% response rate). To the question “What is your primary practice role”—60% responded with obstetrics and 71% with gynecology, and 30% reported both. PAs working in subspecialties included 9% of respondents for urogynecology, 5% for gynecology oncology, 11% for reproductive endocrinology and infertility, 27% for sexual health and gynecology, 2% in maternal-fetal medicine, and 31% for complex family planning.

**FIG. 1.**
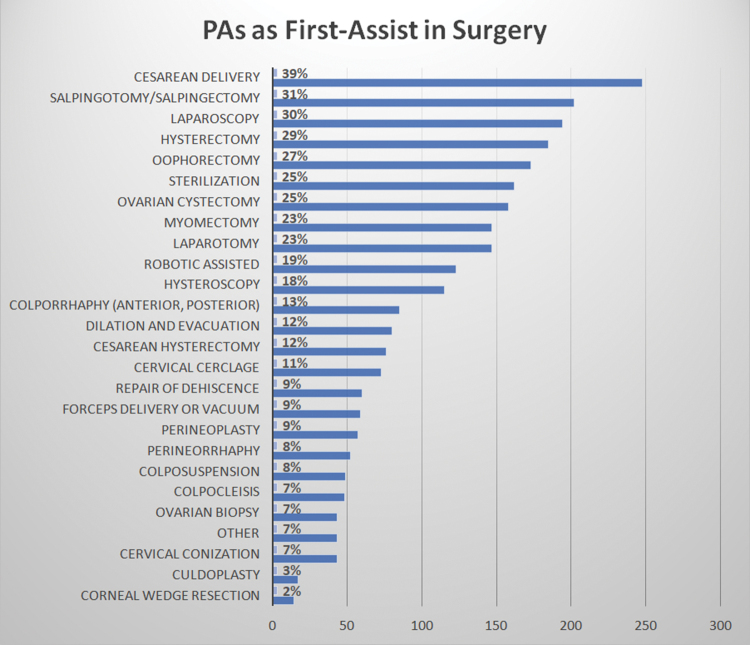
PAs as first-assist in surgery. The types of surgery where PAs assist include Cesarean delivery, salpingotomy/salpingectomy, laparoscopy, hysterectomy, sterilization, ovarian cystectomy, and others. PAs, physician associates.

Most respondents (89%) first assist in OBGyn surgery. The types of surgery where PAs assist include Cesarean delivery (39%), salpingotomy/salpingectomy (31%), laparoscopy (30%), hysterectomy (29%), sterilization (25%), ovarian cystectomy (25%), and others (7%) (Fig. 1). Respondents could choose more than one answer.

**FIG. 2.**
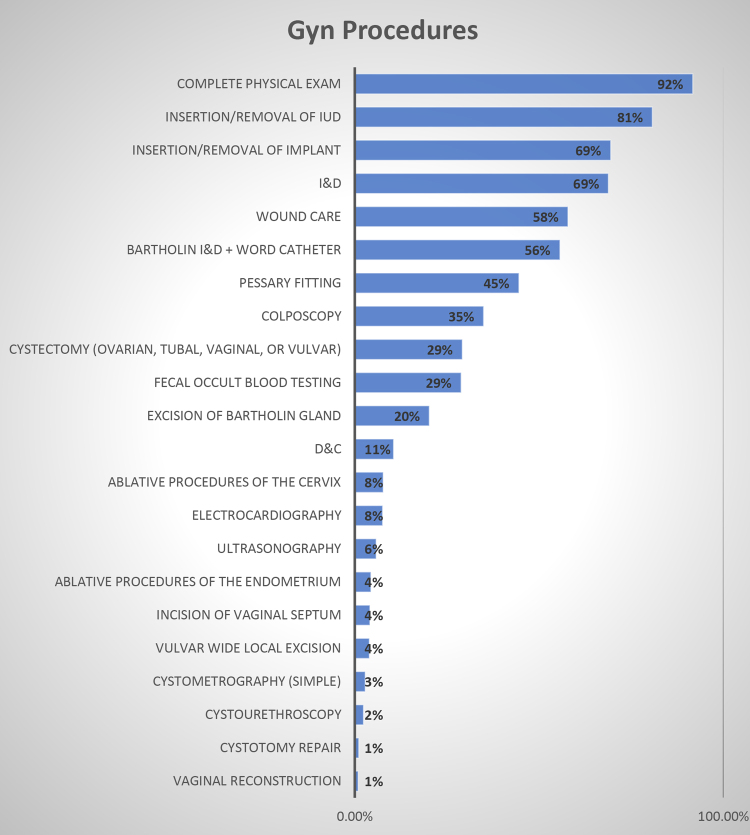
Office-based gynecological procedures by PAs. Most PAs in OBGyn are office-based and perform a complete physical examination. A similar number insert and remove long-acting contraceptive devices, incise and drain cysts, fit pessaries, and perform colposcopies. OBGyn, obstetrics and gynecology.

**FIG. 3.**
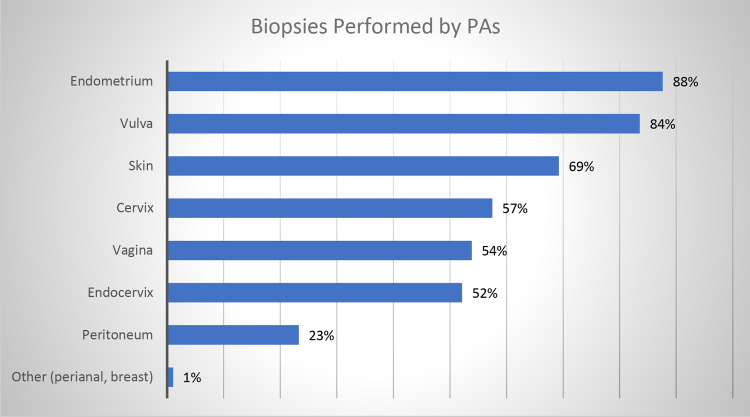
Biopsies performed by PAs in the office. Biopsies include the cervix, endometrium, skin, vagina, vulva, and endocervix.

Most PAs in OBGyn are office-based and perform a complete physical examination (92%). A similar number insert and remove long-acting contraceptive devices (81%), incise and drain cysts (69%), fit pessaries (45%), biopsy (64%), and perform colposcopies (35%) (Fig. 2). More specifically, PAs perform biopsies of the cervix (57%), endometrium (88%), skin (69%), vagina (54%), vulva (84%), and endocervix (52%) to assess for benign and malignant pathology (Fig. 3).

**FIG. 4.**
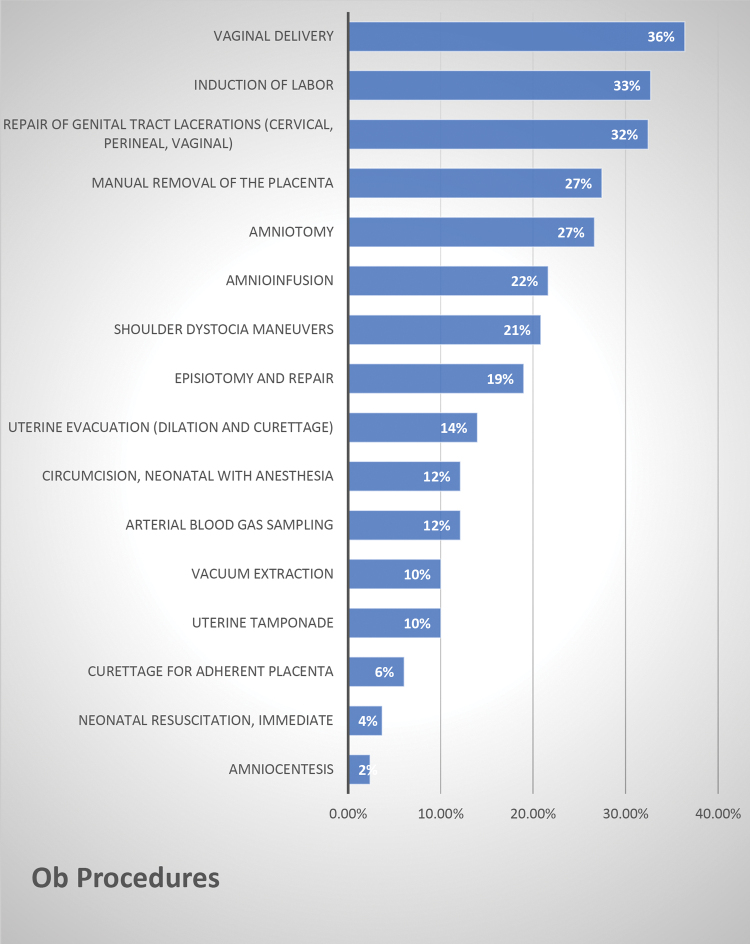
Obstetrical procedures (rank order) reported by PAs.

A majority (59%) of PAs perform an antepartum fetal assessment, and the types surveyed ranged from dating ultrasound 11% to biophysical profile (4%) and nonstress testing (5%). Inpatient obstetric procedures were also queried, and 35% of respondents that answered “yes” performed intrapartum fetal assessment, 17% performed vaginal deliveries, 56% induced labors, 55% repaired genital tract lacerations, and 47% manually removed the placenta when indicated (Fig. 4). Where respondents reported “other,” the comments included mechanical cervical ripening, fetal scalp electrode insertion/removal, intrauterine pressure catheter insertion/removal, and vacuum extraction. Notably, at least 39% of PAs first assist in Cesarean deliveries.

A secondary list included subspecialty procedures. These questions were created as a checklist in the survey (Table 1; Combines Q 24, 25, 27, 31). Regarding abortion services, there were 76 respondents, of which 37% provided medical abortion, 14.5% offered surgical abortion, and 14.5% performed manual vacuum aspiration.

**Table 1.** Combined Four Survey Questions

Types of outpatient procedures		
	Percent of respondents	Total number
Pessaries	58.33	189
Ovulation induction	35.29	60
Paracervical block	16.67	54
Intrauterine insemination	22.94	39
Cerclage removal	48.72	38
Hysterosalpingogram	18.82	32
Urodynamics	8.95	29
Cerclage placement	23.08	18
Amniocentesis	16.67	13
Transvaginal tape procedure	3.40	11
Percutaneous umbilical cord sampling	5.13	4
Chorionic villus sampling	2.56	2

In addition, participants were asked to list procedures that were not included in the survey. The following were additional procedures:

- Intrauterine insemination.
- Laser treatments.
- Pelvic organ prolapse assessment.
- Mental health care and assessments.
- Trigger point injections.
- Trichloroacetic acid application.
- Egg retrievals.
- Mock transfers.
- Saline sonohysterogram.

An open-ended section at the end of the survey invited comments relevant to the study, the role of the PA in OBGyn, or interaction with other members of the women's health team. A thematic analysis of common phrases and summaries is provided (Table 2).

**Table 2.** Thematic Analysis

Theme 1: Women's healthcare providers
“There is confusion about what a Women's Health NP (WHNP), a nurse midwife, and a PA can do in OB/GYN. Midwives can manage low-risk cases. WHNPs can manage a broader range of care *excluding* the active stage of labor. PAs provide a full range of obstetric and gynecologic care including first assisting in surgeries.”
Theme 2: Vaginal deliveries
“Physicians and nurses often have misconceptions about the capabilities of PAs in OB/GYN, which can lead to underutilization. Hospitals often limit the ability of PAs to be credentialed for procedures or deliveries, despite having the necessary training and experience. Many physicians believe that only CNMs can perform vaginal deliveries. Some physicians are reluctant to allow PAs to work independently in OB/GYN due to concerns about billing and malpractice. There is a lack of opportunities for PAs in OB/GYN, particularly in hospital settings. PAs can legally perform normal vaginal deliveries and are often preferred for OBGYN positions. However, some on-the-job training and experience may be needed.”
Theme 3: Privileges
“Hospital OBGYN departments do not often grant privileges to PAs, and some hospital boards limit their access to certain procedures or deliveries.”
Theme 4: Surgeries
“PAs can perform surgical procedures and first assist in more complex surgeries.”
Theme 5: Outpatient
“Some physicians are unsure how to incorporate PAs into their team-based practices, and some may be hesitant at first. Some do not permit them to perform certain procedures, and some do not pay them as much as NPs.”
Theme 6: Acceptance
“Nurses/professional medical team do not understand the role of a PA. There can be discrimination towards PAs in OBGYN.”

## Discussion

This is the first survey about the procedural roles of PAs specialized in women's health. From a baseline of 1,054 PAs in OBGyn, 729 responded to the anonymous survey (47% response rate). Thirty questions were posed with answers that could be marked easily and electronically, intended to simplify participation. Optional text boxes followed each question for clarification or comment. The results present a wide-ranging overview of PAs in this specialized role. What PAs do and where they work is broad.^7^ While the American PA movement is only half a century in development, the range of service delivery of 154,000 certified PAs is emerging as unique, significant, and resourceful.^6^

The rationale for undertaking a procedure study was based on anecdotal observations that PAs may be more procedurally oriented than NPs or CNMs when viewed in the same setting. On top of this, access to obstetrical and gynecological care is declining, which is more significant in underserved populations.^11^ With the modeling of women's health providers by the HRSA, which predicted an ongoing contraction of obstetricians and gynecologists, filling some provider gaps would be by other clinicians.^12^ The same HRSA Report predicted the PA supply in women's health would grow by 56% (from 1,480 to 2,310 full-time equivalents), and CNM and NP supply would increase by 32% and 89%, respectively, by 2030. The work presented here is intended to complement that report and set the stage for a more granular analysis of care outcomes, role delineation, productivity, patient satisfaction, and optimal team use.

There is a rising need and call for improved maternal–child health outcomes in the United States.^13,14^ The *Healthy Starts* initiative provided more than $115 million to fund 101 community undertakings to improve access to perinatal care. This is in response to the nation's maternal mortality rate increasing from 17.4 deaths per 100,000 live births in 2018 to 23.8 deaths in 2020.^15^ The 2021 maternal mortality increased to 32.9 in 100,000 live births. “Maternity care deserts” in the United States are emerging due to childbearing services becoming scarcer and women's health providers leaving areas stranded without clinics and maternity services.^13,15^ On top of this closing of women's health services, a 2017 report by the American College of Obstetricians and Gynecologists (ACOG) states, “The pace of life and its stresses, impact from multitasking, overwhelming information exposure, and electronic medical record expectations have led to some degree of physical or emotional exhaustion or lack of motivation.

Physicians have burnout rates that are twice the rate of other working adults, and no area of medicine is immune.”^16^

Burnout is pervasive across many medical-surgical specialties and is associated with reduced clinical productivity and early retirement.^16^ How this may impact the impending OB/GYN shortage is speculative. One possible solution to workforce issues causing burnout may be to employ more teams of women's health specialists. Evidence is growing that team-based care can alleviate some of the fatigue effects and improve job satisfaction.^17,18^ There are predictions that an increased female OB/GYNs rate will grow to 66% of the workforce by 2030.^19^ The changing roles of obstetrical providers, such as hospitalists, appear to have workforce implications and mitigation strategies that include PAs and NPs.^20^

From this foundation on procedures by PAs, the stage is set to understand better what they do, their productivity, patient satisfaction, job satisfaction, and contributions to team-based care. What are the skills and encounters of PAs in the same role in 18 countries that employ PAs?^21^ How do the activity of PAs and their quality of care compare to physicians, NPs, and CNMs in this field?

### Limitations

This study comprises data from a survey in that almost half of the PAs who once declared they worked in OBGyn were participants. To date, it is the first and most comprehensive national collection of PA workforce activity in this area of specialization. But there are some limitations. First, an e-mail list of self-declared PAs in OBGyn was obtained from the AAPA, which holds historical membership of PAs. The member chooses to update. As a result, not all were clinically active or working in women's health by the time of the survey. This is the leading reason why the response rate is near 50%. Thus, these findings may underestimate the full extent of procedures by certified PAs practicing OBGyn in the United States.

Moreover, PAs in other specialties, such as family medicine and general surgery, who provide care to women were not accounted for in this study. Another limitation is that our research relied on self-reported health workforce data, which have inherent limitations, including misinterpretation of questions and potential recall and acquiescence biases.

This study's strength is in the proceduralist skills that PAs in OBGyn bring to the clinical setting. The list of procedures generated was expansive and new to some readers of this study. Such findings set the stage for understanding where the division of labor occurs in OBGyn service delivery and what the new PA interested in a career in women's health will need to know in this field.

## Conclusion

At the beginning of 2023, 1,054 clinically active PAs reported they worked in OBGyn and provided various procedural services. Among the many roles, they first assist in open, laparoscopic, and robotic surgery along with Cesarean section, hysterectomy, and salpingo-oophorectomy. Subspecialty surgeries included oncology and urogynecology. In the outpatient setting, the range of gynecological procedures was at least 40. The list included biopsies of the endometrium, cervix, vagina, vulva, other lesions, fetal assessment, ultrasonography, insertion and removal of devices, and other procedures. While this medical group has not been well delineated in the literature, newer information suggests that PAs in women's health represent a needed provider during the increasing demand for specialists in childbearing medicine.

## Abbreviations Used

AAPA: Academy of Physician Associates
APAOG: Association of Physician Associates in Obstetrics and Gynecology
CNM: certified nurse midwives
HRSA: *Health Resources and Services Administration*
PA: physician associate
NCCPA: National Commission on the Certification of PAs
NP: nurse practitioner
OBGyn: obstetrics and gynecology
